# Honokiol attenuates the severity of acute pancreatitis-associated lung injury by acceleration of acinar cell apoptosis

**DOI:** 10.1186/cc9674

**Published:** 2011-03-11

**Authors:** T Weng

**Affiliations:** 1 National Taiwan University, Taipei, Taiwan

## Introduction

Acute pancreatitis (AP) is a complicated immunological response that leads to multiple organ failure. Apoptosis is a beneficial form of cell death in AP. Acute lung injury is the most severe complication. Honokiol (HK) is a component of Asian herbal teas. It displays an anti-inflammatory and apoptotic induction effect. In the experiments, we investigated the therapeutic efficacy of HK in AP.

## Methods

Adult BALB/c mice were divided into one control and five AP groups. Mice received six injections of cerulein at 1-hour intervals then on intraperitoneal injection (i.p.) of LPS for the induction of AP. Mice in the other groups had injections of cerulein and LPS as described above, but also received an i.p. of the different doses of HK 10 minutes after the first cerulein injection. Cytokine levels for the early and late inflammatory markers were obtained at 3 hours and 24 hours after the end of experiments.

## Results

**Figure 1:**
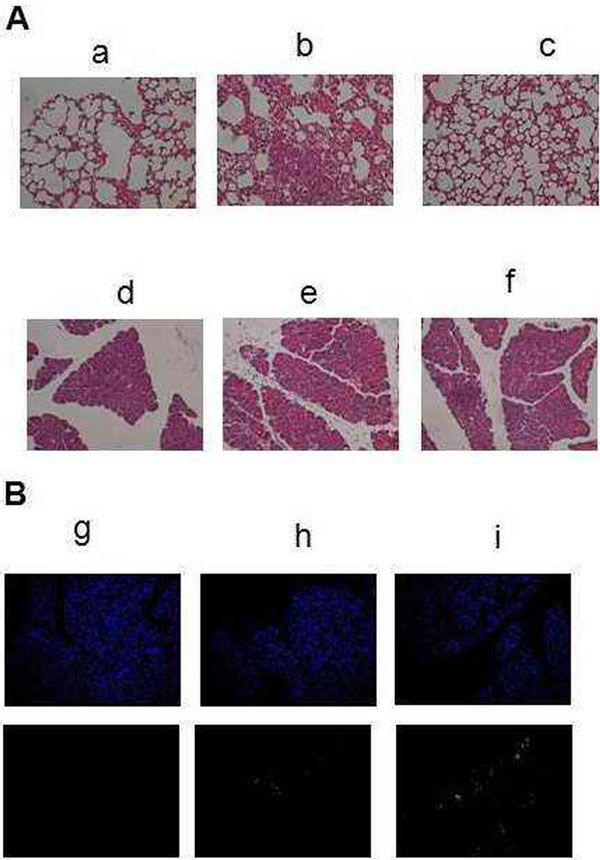


HK protected against the severity of AP in serum amylase/lipase, TNFα, IL-6, HMGB1, and pancreas and lung pathological injury (Figure 1A). Acinar cell apoptosis was increased in the pancreas. Treatment with HK caused markedly increased acinar cell apoptosis (Figure 1B).

## Conclusions

HK attenuates the severity of AP and lung injury by acceleration of acinar cell apoptosis.
